# Supplementary material for: Definite photon deflections of topological defects in metasurfaces and symmetry-breaking phase transitions with material loss
Source: Nat Commun. 2018 Oct 15;9:4271. doi: 10.1038/s41467-018-06718-9 (PMC6189048; doi:10.1038/s41467-018-06718-9)
Supplement: Supplementary file 1 — Supplementary Information [file 41467_2018_6718_MOESM1_ESM.pdf]

Supplementary Materials

Definite Photon Deflections in Topological Metasurface-engineered waveguides and Symmetry Breaking Phase Transitions with Material Loss

Sheng et al.

Supplementary Note 1. The effective medium of topological Space of Cosmic String

We start with a spacetime metric for a static cylindrically symmetric cosmic string with straight and infinite long length:

$$\mathrm{d}s^2 = \mathrm{d}t^2 - \mathrm{d}r^2 - \alpha^2 r^2 \mathrm{d}\varphi^2 - \mathrm{d}z^2 \tag{1}$$

where $\alpha = 1 - 4G\mu$, G is the gravitational constant, μ is the linear mass density of the string along the rotation axis, and the nature units have been adopted. The corresponding Riemann curvature is given by $R^{12}_{12} = 2\pi(1-\alpha)\delta^{(2)}(r)/\alpha$, where $\delta^{(2)}(r)$ is the 2-dimedional Dirac delta function in the plane. Therefore, the string is locally flat with a conical singularity at the origin. According to Riemann curvature, if the mass density of the string $\mu > 0$, then it carries positive curvature; on the other hand, it carries negative curvature with negative mass density ($\mu < 0$). It is well known that the motion of photon follows the null geodesic of the space-time, thus the trajectory of light move toward (away) to the origin of the string with positive (negative) curvature. It is analogous to that the charged particle moves under the circumstance of point charge. According to geodesic equation: $\ddot{x}^\lambda + \Gamma^\lambda_{uv}\dot{x}^u\dot{x}^v = 0$, where x is spatial coordinates and the derivatives are taken over an arbitrary affine parameter. For metric of the string, it has nonzero Christoffel symbols $\Gamma^{\mathrm{r}}_{\varphi\varphi} = -\alpha^2 r$, $\Gamma^{\varphi}_{\mathrm{r}\varphi} = \Gamma^{\varphi}_{\varphi\mathrm{r}} = 1/r$, and the light trajectory after some calculation can be described by

$$r^2\alpha^2\dot{\varphi}^2 = C\dot{r}^2 \tag{2}$$

where $C = b^2/(\rho^2 - b^2)$, b is impact parameter. Therefore, we can numerically calculate the trajectory and deflection angle of light motion under different impact parameter. Furthermore, with a new angular coordinate $\phi = \alpha\varphi$, the metric takes a Galilean form $\mathrm{d}s^2 = \mathrm{d}t^2 - \mathrm{d}r^2 - \rho^2\mathrm{d}\phi^2 - \mathrm{d}z^2$. The metric, however, does not describe a Euclidean space, since ϕ changes from 0 to $\alpha \cdot 2\pi$. The motion of the photon in this coordinate follows straight lines. As the radial coordinate changes from a very large distance ($R \gg r$) to the point closest (r_0) of the string and again to the very large distance R, the corresponding

angle in ϕ is $\delta\phi = \pi$, and $\delta\varphi = \pi/\alpha$. Thus the light deflection angle is $\Delta\theta = \delta\varphi - \pi = \pi(1-\alpha)/\alpha$ and is independent of the impact parameter.

To realize effective parameter required by cosmic string, the calculation based on the correspondence between material electromagnetic parameters and the metric of spacetime in general relativity according to transformation optics:

$$\varepsilon^{ij} = \mu^{ij} = \sqrt{-g}g^{ij} / \left(\sqrt{\zeta}g_{00}\right) \tag{3}$$

where $\varepsilon^{ij}$and μ^{ij} are artificial material parameters, g^{ij} is the metric of the cosmic string based on Supplementary equation (1), ζ is determinant of a spatial metric to transform one set of spatial coordinates to another. Such tensors have their principal values along r, φ and z directions:

$$\varepsilon_{\mathrm{r}} = \mu_{\mathrm{r}} = \alpha,\ \varepsilon_{\varphi} = \mu_{\varphi} = 1/\alpha,\ \varepsilon_{\mathrm{z}} = \mu_{\mathrm{z}} = \alpha \tag{4}$$

which can also be obtained by performing a linear transformation along the φ direction from a Minkovski space $\mathrm{d}s'^2 = \mathrm{d}t'^2 - \mathrm{d}r'^2 - r'^2\mathrm{d}\varphi'^2 - \mathrm{d}z'^2$: $r' = r, \varphi' = \alpha\varphi, z' = z$. Here, considering different polarized light, we can define two orthogonal polarized waves: transverse electric (TE) wave with fields $(E_{\varphi}, E_{\mathrm{r}}, H_{\mathrm{z}})$ and transverse magnetic (TM) wave with fields $(H_{\varphi}, H_{\mathrm{r}}, E_{\mathrm{z}})$. For TE wave, we can take the refractive index $\left(n_{\varphi}^2 = \epsilon_{\mathrm{r}}\mu_{\mathrm{z}} = \alpha^2, n_{\mathrm{r}}^2 = \epsilon_{\varphi}\mu_{\mathrm{z}} = 1\right)$. For TM wave, we can take the refractive index $\left(n_{\varphi}^2 = \mu_{\mathrm{r}}\epsilon_{\mathrm{z}} = \alpha^2, n_{\mathrm{r}}^2 = \mu_{\varphi}\epsilon_{\mathrm{z}} = 1\right)$. Thus, for both two polarizations, the corresponding refractive indexes of the cosmic string are equivalent to a uniaxial crystal with a rotating axis $n = \begin{pmatrix} n_{\varphi} & 0 \\ 0 & n_{\mathrm{r}} \end{pmatrix}$.

Supplementary Note 2. Effective index of artificial waveguides

In Supplementary Figure 1(a), a slab waveguide structure is shown, made of Air/PMMA/Silver/SiO2 multilayer. The light is transported inside the PMMA layer. Firstly, the effective refractive index of the waveguide mode can be obtained by calculating the dispersion relation of waveguide mode propagation constant. For TE (TM) waves, the dispersion relationship are given as:

$$\exp[2ik_2 d] = \frac{1 + r_{32}r_{43}\exp[2ik_3 t]}{r_{12}r_{32} + r_{12}r_{43}\exp[2ik_3 t]} \qquad (5)$$

where $r_{ik} = (\eta_k - \eta_i)/(\eta_k + \eta_i)$ are the reflection coefficients, t is the metal layer thickness, the specific impedances for TE wave and TM wave are respectively $\eta_\mathrm{i} = \sqrt{n_\mathrm{i}^2 - n_\mathrm{nef}^2}$ and $\eta_\mathrm{i} = \sqrt{n_\mathrm{i}^2 - n_\mathrm{nef}^2/n_\mathrm{i}^2}$, and transversal wave vectors is $k_\mathrm{i} = (\omega/c)\sqrt{n_\mathrm{i}^2 - n_\mathrm{nef}^2}$, where n_nef is the effective waveguide index and n_i are the refractive index of each media ((1)-air, (2)-PMMA, (3)-Silver, and (4)-SiO_2). Note that Supplementary Equation. (5) cannot be solved explicitly with respect to the effective index but it can be solved with respect to the film thickness

$$d = \frac{1}{k_2}\left(m\pi + \frac{1}{2i} ln\left(\frac{1 + 1 + r_{32}r_{43}\exp[2ik_3 t]}{r_{12}r_{32} + r_{12}r_{43}\exp[2ik_3 t]}\right)\right) \qquad (6)$$

where m is the mode order number. In the calculations, the parameters are n(Air)=1.0, n(SiO_2)=1.55, n(PMMA)=1.49+iγ, and at the operation wavelength λ=785nm, the silver index n(silver) =0.042+i5.309. Finite element software COMSOL Multiphysics is used to retrieve effective index of the waveguide modes. Supplementary Figure 1(b) show the calculated iso-frequency contour of the first order (m=1) transverse electric (TE) and transverse magnetic (TM) waveguide modes, which are both circular curves. That means the slab waveguide has isotropic effective index distribution. Supplementary Figure 1(c) shows the dependence of effective index of TE mode and TM mode on PMMA layer thickness.

In order to obtain anisotropic effective index in the artificial waveguide, we replace the silver layer with a metasurface consisting of a subwavelength grating (see Supplementary Figure 1(d) and the inset), which is employed to weakly disturb the slab modes. The

calculated effective TE and TM mode indexes are given in Supplementary Figure 1(e). Due to metasurface, the slab waveguide modes are weakly disturbed and changed to anisotropic modes. Correspondingly, the iso-frequency contours are changed from circles to ellipses, as shown in Supplementary Figure 1(e). The anisotropic indices n_e and n_o are retrieved, which is equivalent to a uniaxial crystal. For TE mode, it is analogous to a positive uniaxial crystal material with $n_\mathrm{e} > n_\mathrm{o}$, while for TM mode, it is analogous to a negative uniaxial crystal material with $n_\mathrm{e} < n_\mathrm{o}$. Supplementary Figure 1(f) shows the thickness dependence of the effective index of TE mode and TM mode.

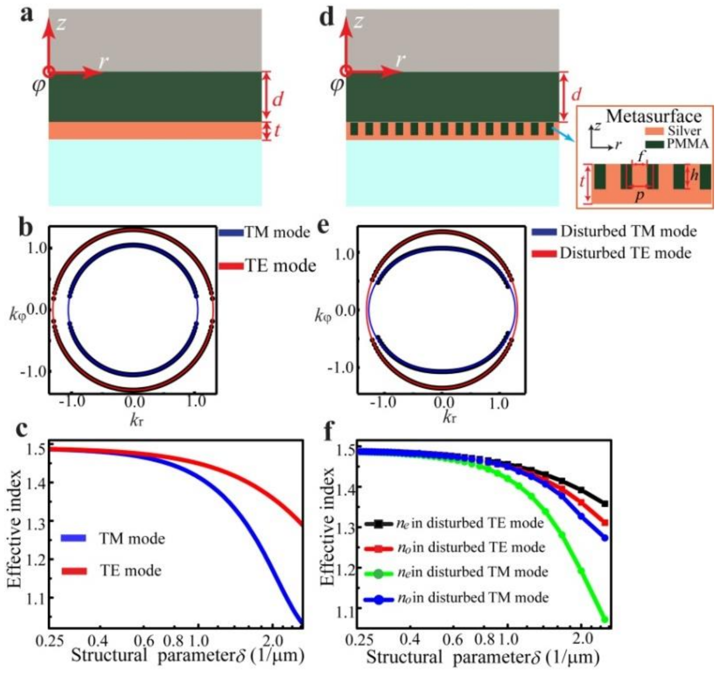

Supplementary Figure 1. The effective index of waveguide modes: (a) The schematic of a slab waveguides which consists SiO_2/Silver/ PMMA/Air multilayer structure: the bottom cyan layer indicates silica (SiO_2) substrate, the orange layer is silver layer, the green layer is PMMA, and top light gray lay is air. In order to describe circular metasurface, a cylindrical coordinate (z, r, φ) is taken.The used parameters are $d = 400\mathrm{nm}, t = 200\mathrm{nm}$. (b) The iso-frequency contour of TE and TM mode of slab waveguide. The dots are retrieved from COMSOL software; and the red solid lines are fitted curves based on the formula $(k_\mathrm{r}/n_\mathrm{c})^2 + (k_\varphi/n_\mathrm{c})^2 = 1$, where $n_\mathrm{c}^\mathrm{TE} = 1.305$ for TE mode, and $n_\mathrm{c}^\mathrm{TM} = 1.054$ for TM mode, k_r and k_φ are respectively propagation wave vectors along the radial and azimuthal direction (c) The thickness $(\delta = 1/d)$ dependence of effective index of TE mode and TM mode of the slab waveguide. (d) The schematic of artificial waveguide based onSiO_2/Silver/Metasurface/PMMA/Air multilayer structure, with the parameters $p = 120nm$, $d = 400\mathrm{nm}, t = 200\mathrm{nm}, h = 60\mathrm{nm}$, and the silver filling ratio $f = 0.58$. (e) The iso-frequency contours of disturbed TE and TM modes. The dots are retrieved from COMSOL; and the red solid lines are fitted curve based on the formula $(k_\mathrm{r}/n_\mathrm{o})^2 + (k_\varphi/n_\mathrm{e})^2 = 1$, and $n_\mathrm{o}^\mathrm{TE} = 1.311$, $n_\mathrm{e}^\mathrm{TM} = 1.358$ for TE mode; $n_\mathrm{o}^\mathrm{TM} = 1.274$, $n_\mathrm{e}^\mathrm{TM} = 1.072$ for TM mode. (f) The thickness $(\delta = 1/d)$ dependence of the effective index of distributed TE and TM modes of the artificial waveguide.

Supplementary Note 3. Topological Phase transition points under different material loss

In our model, we can include material loss through defining the imaginary part of refractive index of PMMA $n(\text{PMMA}) = 1.49 + i\gamma$. Here, the loss can be introduced by doping quantum dots inside the PMMA layer in spin-coating process. The loss coefficient γ can be changed through changing the doping solubility. After including the material loss, we recalculate the effective index of extraordinary waveguide modes n_{e}. With different doped level of quantum dot in PMMA dielectric layer, the loss parameter γ has different value. And the propagation length can be calculated as $l_{\mathrm{TE(TM)}} = \frac{1}{2\cdot \mathrm{Im}\left(n_{\mathrm{nef}}^{\mathrm{TE(TM)}}\right)\cdot k_0}$, where $k_0 = 2\pi/\lambda$, just as shown in Supplementary Figure 2(a). The higher doped level has smaller propagation length. Due to the uncertainty relation of position and momentum $\Delta x \Delta p \geq \hbar/2$, where propagation momentum is $p = \hbar n_{\mathrm{nef}} k_0$, and the propagation length is $\Delta x = l_{\mathrm{d}} = \max(l_{\mathrm{TE}},\ l_{\mathrm{TM}})$ in the waveguide. So if we can make a distinction between two modes, this requires the propagation coherence length $l_{\mathrm{coh}} = 2\pi\hbar/\Delta p < 4\pi l_{\mathrm{d}}$. Here, we define the critical length $l_{\mathrm{c}} = l_{\mathrm{coh}}/4\pi$. Then we can conclude, when the propagation loss length is smaller than the critical length $l_{\mathrm{d}} < l_{\mathrm{c}}$, the two modes cannot be differentiated from each other.

For metasurface waveguide, the ellipticity discrepancy between distributed TE mode and distributed TM mode is $\Delta\eta = \eta^{\mathrm{TE}} - \eta^{\mathrm{TM}} = n_{\mathrm{e}}^{\mathrm{TE}}/n_{\mathrm{o}}^{\mathrm{TE}} - n_{\mathrm{e}}^{\mathrm{TM}}/n_{\mathrm{o}}^{\mathrm{TM}}$. The numerically calculated results from finite element software COMSOL Multiphysics clearly show that for larger dielectric thickness $n_{\mathrm{o}}^{\mathrm{TE}} \approx n_{\mathrm{o}}^{\mathrm{TM}} = n_{\mathrm{o}}$ (see Supplementary Figure 1(f)), then ellipticity discrepancy can be simplified as $\Delta\eta = \left(n_{\mathrm{e}}^{\mathrm{TE}} - n_{\mathrm{e}}^{\mathrm{TM}}\right)/n_{\mathrm{o}}$. Supplementary Figure 2(b) shows the critical length $l_{\mathrm{c}} = \hbar/2\Delta p = 1/\left(2k_0 \cdot \left(n_{\mathrm{e}}^{\mathrm{TE}} - n_{\mathrm{e}}^{\mathrm{TM}}\right)\right) = \lambda/\left(4\pi\left(n_{\mathrm{e}}^{\mathrm{TE}} - n_{\mathrm{e}}^{\mathrm{TM}}\right)\right)$ and propagation length l_{TE} and l_{TM} respectively for TE mode and TM mode in the waveguide. And there exist critical thickness parameter δ_{c} for different loss parameter γ. If taking loss parameter $\gamma = 0.0015$ as an example, the corresponding critical thickness parameter is $\delta_{\mathrm{c}} = 1/d_{\mathrm{c}} = 0.335$. If the dielectric thickness parameter is smaller than δ_{c}, the coherence propagation length l_{c} for distributed TE mode and distributed TM mode is larger than the propagation length caused by loss, then two modes cannot make a distinction (just shown left zone in Supplementary Figure 2(a)). Therefore, the intersection point δ_{c} can be seen as the critical phase transition point between trivial symmetric modes and nontrivial symmetric

breaking modes. Also, the transition parameter δ_{c} can also be tuned by loss parameter γ in the PMMA dielectric layer (just shown dashed dot green line in Supplementary Figure 2(a)) . Supplementary Figure 2(b) shows the relation between the transition parameter δ_{c} and loss parameter γ in the trasnformation optical waveguide.

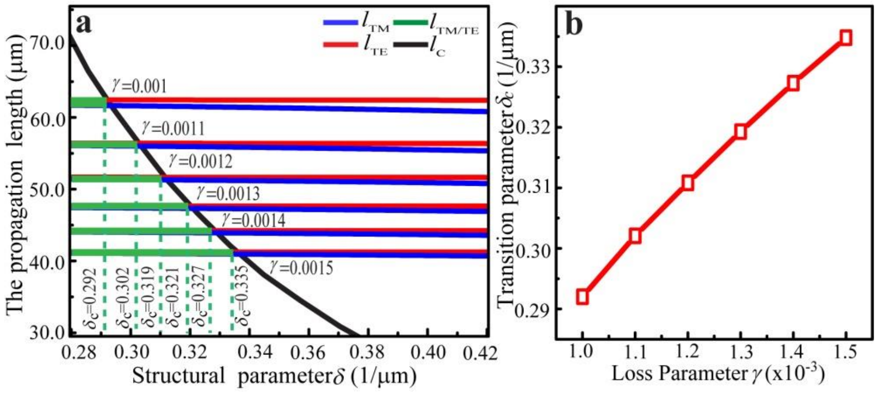

Supplementary Figure 2. **The propagation length and coherence propagation length of waveguide modes**: (a) The thickness dependence of critical length l_{c} and propagation length l_{TE} and l_{TM} under different material loss γ. And with higher doped level of quantum dot, the waveguide has larger γ and smaller propagation length. After comparing critical length l_{c} with propagation length $l_{\mathrm{d}} = \max(l_{\mathrm{TE}}, \ l_{\mathrm{TM}})$, there exist critical structural parameter δ_{c} for different loss γ. (b) The dependence of transition parameter δ_{c} on loss parameter γ.
