# Supplementary material for: Definite photon deflections of topological defects in metasurfaces and symmetry-breaking phase transitions with material loss
Source: Nat Commun. 2018 Oct 15;9:4271. doi: 10.1038/s41467-018-06718-9 (PMC6189048; doi:10.1038/s41467-018-06718-9)
Supplement: Supplementary file 3 — Description of Additional Supplementary Files [file 41467_2018_6718_MOESM3_ESM.pdf]

Description of Additional Supplementary Files

File Name: Supplementary Movie 1

Description: The movie shows with increasing the structural parameter δ, there exists critical phase transition point δ_c between trivial symmetric phase and nontrivial symmetric breaking phase. Through the critical point, the TE and TM modes will be transformed from the degenerated modes (see white dashed circle iso-frequency contour in figure(a) and figure (b)) to the degeneracy breaking modes (see elliptical iso-frequency contour $\eta_{TE} > 1$ in figure (a) and $\eta_{TM} < 1$ in figure (b)). Definite photon deflection in topological negative space mimicked by TE mode (c) and positive space mimicked by TM mode (d).
